# Long-term alterations in pain sensitivity following preterm birth: a systematic review and meta-analysis

**DOI:** 10.3389/fped.2026.1858823

**Published:** 2026-07-08

**Authors:** Jorge Sánchez-Infante, Juan Manuel Pérez-Pozuelo, Almudena Crespo-Cañizares, Sonsoles Hernández-Iglesias, Jara Esteban-Sopeña, Ana Sánchez-Tovar, Sagrario Gómez-Cantarino, Miriam Hermida-Mota, Nuria García-Magro

**Affiliations:** 1 Institute of Health and Sport Sciences, Faculty of Health Science, Universidad Francisco de Vitoria, 28223, Madrid, Spain; 2 Grupo de Investigación en Fisioterapia Toledo (GIFTO), Facultad de Fisioterapia y Enfermería, Universidad de Castilla-La Mancha, Toledo, Spain; 3 Grupo de Investigación en Enfermería Toledo (CuidArte-Diputo), Faculty of Physiotherapy and Nursing, University of Castilla-La Mancha, Toledo, Spain; 4 Faculty of Health Sciences, Department of Nursing, Universidad Francisco de Vitoria, Pozuelo de Alarcón, Madrid, Spain

**Keywords:** childhood, long-term outcomes, pain sensitivity, pain threshold, preterm

## Abstract

**Background and objective:**

Preterm infants undergo repeated painful procedures during neonatal care, potentially leading to long-lasting alterations in nociceptive processing. Evidence on later pain sensitivity remains inconsistent. This study synthesised long-term evidence on thermal and mechanical pain thresholds and pain intensity from school age to young adulthood in individuals born preterm vs. term.

**Databases and data treatment:**

A systematic search of PubMed, Cochrane Library, Web of Science and Scopus was conducted up to September 2025. Observational studies including preterm- and term-born participants aged 7–28 years were eligible. Outcomes included self-reported pain intensity and quantitative sensory testing (QST) measures of thermal and mechanical pain and detection thresholds. Random-effects meta-analyses used standardised or mean differences; heterogeneity was quantified with *I*^2^.

**Results:**

Eight studies (731 participants; 328 preterm, 403 term-born) contributed 2,237 participant–outcome observations (1,140 preterm, 1097 term-born). Preterm-born participants reported higher pain intensity (standardized mean difference = 0.45, *p* = 0.03) and higher heat pain thresholds (mean difference = 1.11 °C, *p* = 0.002) than term-born. No significant differences were observed for other QST outcomes. Several analyses showed substantial heterogeneity.

**Conclusion:**

Preterm birth may be associated with differences in specific pain-related outcomes from childhood through young adulthood, particularly higher heat pain thresholds and pain intensity ratings. Well-designed longitudinal studies with standardized protocols are needed to inform neonatal pain management.

**Systematic Review Registration:**

https://www.crd.york.ac.uk/PROSPERO/view/CRD420251144728, PROSPERO CRD420251144728.

## 1 Introduction

The neonatal period, defined as the first four weeks of life, represents a critical stage in human development marked by rapid brain growth, maturation, and profound plasticity (1, 2). Exposure to early-life stressors, including pain, medical interventions, separation from caregivers, or adverse environmental conditions, can lead to long-lasting physiological and psychological consequences (3, 4). This neonatal period is characterized by rapid synaptogenesis, the establishment of neural circuits, and the refinement of sensory and regulatory systems (2). Given the accelerated nature of these processes, nociceptive or stressful disruptions, may interfere with the organization of neural pathways that will later support complex functions such as attention, memory and sensory discrimination (5–7).

These exposures are particularly common among preterm-born neonates requiring NICU care. Advances in neonatal care have significantly improved survival rates, among preterm-born infants; however, this increased survival is often accompanied by repeated exposure to invasive and painful medical procedures during critical periods of neurodevelopment (8, 9). Premature infants frequently undergo numerous painful interventions throughout hospitalization (9–13) and, unlike older children and adults, they have immature inhibitory pathways and heightened excitatory responses, making them particularly vulnerable to alterations in pain processing (14–16). Moreover, cumulative nociceptive exposure, combined with prolonged hospitalization, may further amplify long-term effects on neural organization and sensory processing (13).

Evidence from human and animal studies indicates that early-life pain may induce long-term alterations in pain sensitivity (4, 17, 18), leading to hypoalgesia or hyperalgesia (19, 20). In humans, repeated neonatal pain exposure can disrupt the neurobiological mechanisms responsible for pain modulation (18). These alterations may affect the reactivity of the autonomic nervous system and the hypothalamic–pituitary–adrenal axis (21, 22). Structural and functional changes have been reported in brain regions involved in pain perception and regulation, including the amygdala, thalamus, and prefrontal cortex (4, 23, 24). Consistently, former preterm-born children often display altered somatosensory function, abnormal nociceptive responses, and differences in pain thresholds compared with term-born (25, 26). Moreover, these differences are not limited to infancy but may persist from childhood into adolescence (27, 28) though underlying mechanisms remain poorly understood.

Building on this evidence, this systematic review and meta-analysis synthesize retrospective observational cohort studies comparing pain thresholds in preterm-born individuals and at term. Our aim is to investigate whether sensory differences during childhood and young adulthood exist as a reflection of the possible long-term consequences of prematurity.

## 2 Materials and methods

This systematic review and meta-analysis was conducted in accordance with the MOOSE (Meta-analysis of Observational Studies in Epidemiology) reporting guidelines (29) and the Preferred Reporting Items for Systematic Reviews and Meta-Analyses (PRISMA 2020) statement (30). The review was registered in the PROSPERO International Prospective Register of Systematic Reviews with the reference number: CRD420251144728.

### 2.1 Data sources and searches

A search was conducted in four databases (PubMed, Cochrane, Web of Science and Scopus) for retrospective observational cohort study, with no time restrictions, from inception to September 2025. The search equations employed were formulated by combining MeSH terms and relevant keywords using the Boolean operators AND and OR. The initial search strategy was developed for PubMed using MeSH terms and free-text terms and was subsequently adapted to the syntax and indexing characteristics of each database. For Scopus, Cochrane and Web of Science, equivalent keyword-based strategies were used (Supplementary Table S1).

To reduce bias, two reviewers (N.G-M. and A.C-C.) conducted independent searches, following a methodology they had jointly established for developing the search equations. Any disagreements were resolved through the involvement of a third researcher (S.H.I.), who facilitated consensus.

### 2.2 Study selection

The selection of studies was based on the criteria established by the PECO(S) framework and the reporting followed the MOOSE checklist (29). (P—Participants: Individuals aged 7–28 years (from childhood through young adulthood) who were born preterm (<37 weeks) or at term (≥37 weeks); E-Exposure: Exposure to prematurity with associated neonatal factors reported (invasive procedures, stress, NICU care); C: Term-born participants without exposure to prematurity; O: Pain thresholds and potential alterations in pain perception from childhood through young adulthood.

Additionally, studies were considered eligible if they were published in either English or Spanish. The exclusion criteria included systematic reviews, meta-analyses, opinion articles, publications available only in abstract form, and duplicate records.

### 2.3 Data extraction

The data extraction process was carried out independently by two reviewers (N.G-M and J.S-I). Full-text versions of all selected studies were retrieved, and the relevant information was organized and summarized in a detailed table. Any disagreements or inconsistencies were addressed and resolved by a third reviewer (A.C-C). When key information was not available in the published manuscripts, the corresponding authors were contacted to request the missing data. When continuous data were reported as medians and interquartile ranges, means and standard deviations were estimated using the method Luo et al. and Wang et al. (31, 32).The following data were extracted: author information, year of publication, country where the study took place, sample details (size, age at which the study was conducted). Assessment of Risk of Bias.

The assessment of bias risk was conducted following the Cochrane guidelines (33)(31), using the ROBINS-I tool designed for non-randomized studies of exposures. Two independent reviewers (J.S-I and N.G-M) conducted the evaluations, and any discrepancies were resolved through discussion or consultation with a third reviewer (A.C-C). The ROBINS-I tool evaluates seven domains of potential bias, including confounding, selection of participants, classification of exposure, deviations from intended exposure, missing data, measurement of outcomes, and selection of reported results. Each domain was rated as having low, moderate, serious, or critical risk of bias, or no information when data was insufficient. To be considered at low overall risk of bias, a study had to be rated as low risk across all domains. If all domains were judged as moderate, the study was considered to have moderate risk. The presence of at least one domain with a serious rating led to a classification of serious risk, while a critical rating in any domain resulted in a designation of critical risk of bias.

### 2.4 Data synthesis and statistical analysis

Only studies that measured pain intensity, heat and cold pain thresholds, mechanical pain, heat detection thresholds, and cold detection thresholds, and that also provided the data necessary for meta-analysis, were included. Six meta-analyses were performed based on the measurements of the different studies. The meta-analysis and statistical analyses were performed using Review Manager software (RevMan, version 5.3; The Nordic Cochrane Centre, The Cochrane Collaboration, Copenhagen, Denmark; 2014). Mean differences (MDs) were used when outcomes were assessed using the same scale and unit of measurement across studies, allowing the pooled effect to be expressed in the original units. Standardized mean differences (SMDs) and 95% confidence intervals were used for continuous outcomes assessed using different scales, units, testing procedures, or reporting formats, allowing comparisons across studies (34). The direction of effect estimates was checked and harmonized before pooling, with effects calculated consistently as preterm-born participants minus term-born participants. The random-effects model was used to account for variability in the effect estimates across studies (34). The heterogeneity among studies was examined using the I² statistic. According to Cochrane recommendations, values between 0% and 40% are generally interpreted as indicating little to no important heterogeneity. Values in the range of 30% to 60% are typically viewed as reflecting moderate heterogeneity. When I² falls between 50% and 90%, this is considered substantial heterogeneity, while values from 75% to 100% indicate considerable heterogeneity (35). Following Cohen's guidelines (36), SDs of 0.2, 0.5, and 0.8 represent small, medium, and large effect sizes, respectively. The significance criterion was set at *p* < 0.05. Assessment of publication bias was not performed for outcomes including fewer than ten studies, as recommended by current methodological guidelines (37). The certainty of evidence for each meta-analysed outcome was assessed using the GRADE framework. The assessment considered risk of bias, inconsistency, indirectness of evidence, imprecision, and publication bias. Publication bias was not assessed when fewer than ten studies were available for an outcome. The GRADE assessment is presented in Supplementary Table S2.

## 3 Results

### 3.1 Study selection

**Figure 1:**
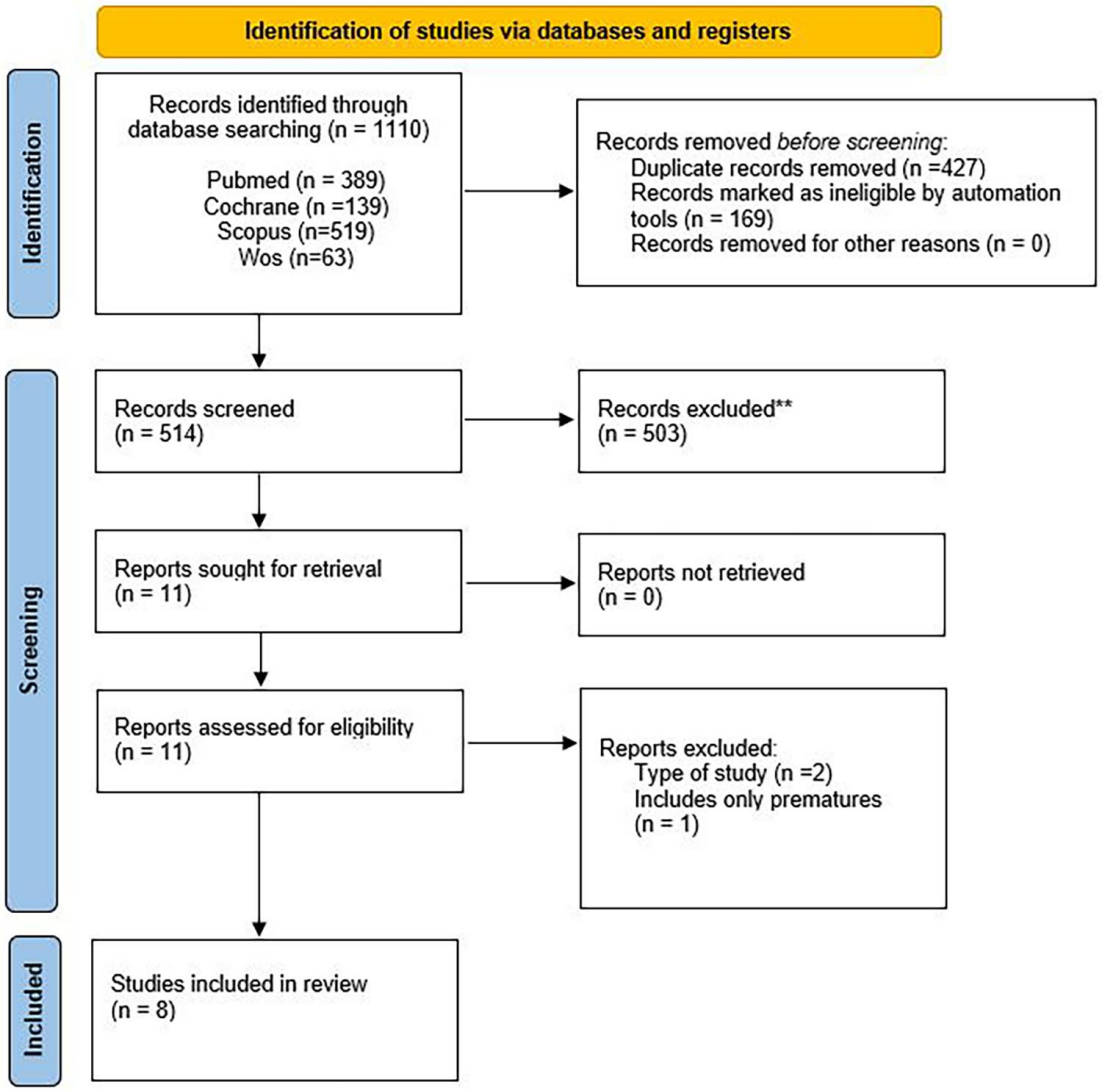
Flow diagram illustrating the study selection process for the present systematic review and meta-analysis.

After screening 1,110 records, a total of eleven full-text articles were selected for detailed assessment. After assessing the full-texts, only eight studies met the inclusion criteria established for this systematic review and meta-analysis. Figure 1 illustrates the study selection process and the reasons for exclusion.

### 3.2 Study characteristics

All included studies examined differences in pain thresholds between preterm-born individuals and those born at term, from childhood through young adulthood. Eight studies including 731 unique participants were included (328 preterm, 403 term-born). Across outcomes, a total of 2,337 participant-outcome observations were analysed (1,140 preterm; 1,197 term-born), with ages ranging from 7 to 28 years. The studies were conducted in several countries, including Norway (38, 39), Canada (40), Israel (41), United Kingdom (42, 43) and Germany (26, 44). The sample sizes across these studies ranged from 18 to 203 individuals, with an average of approximately 88 participants per study. All studies were retrospective observational cohort (26, 39, 40, 42–44), except one case–control observational study (41) and one prospective observational cohort study (38). Pain thresholds were assessed using quantitative sensory testing (QST) methods, including pressure algometry (38, 41, 43), thermal stimulation (38, 39, 42–44), tactile detection thresholds (38, 40, 42–44). The tests were performed under controlled laboratory conditions, with participants indicated the first perception of temperature (detection) and the point at which the sensation became painful (pain thresholds). The main characteristics of the studies are summarized in Table 1.

**Table 1:** Characteristics and design of the included studies.

**Study**	**Design**	**Country**	**Sample size**	**Pain Threshold Assessment**	**Period of Assessment**	**Body region**
Vederhus et al. (39)	Retrospective observational cohort study	Norway	*N* = 59 (31 preterm GA: 26.8 ± 1.8 weeks; 28 term-born)	CPT (^o^C)	17.8 years	right hand to the level of the wrist
Goffaux et al. (40)	Retrospective observational cohort study	Canada	*N* = 26 (13 preterm GA: 29.5 ± 2.2 weeks; 13 term-born)	Thermal test: HPT(^o^C), Conditioning stimulus CPT (^o^C),	7–11 years	left calf and left forearm
				Suprathreshold Heat Pain Intensity (VAS, 0–10)		
Buskila et al. (41)	Case-control observational study	Israel	*N* = 120 (60 preterm GA: 31.4 ± 2.1 weeks; 60 term-born)	Dolorímeter	12–18 years	9 tender points-sites and 4 control point sites: Trapezius, occiput, anterior aspect of intertransverse space at C5–7, second costochondral junction, medial knees, lateral elbow, greater trochanter, forehead, forearm, lateral knee, shaft of the third metatarsal
Walker et al. (42)	Retrospective observational cohort study	United Kingdom	*N* = 87 (43 preterm GA: 24.6 ± 0.67 weeks; 44 term-born)	Thermal test: CPT (^o^C), HPT (^o^C) Mechanical test: MDT(vFh), Brush allodynia (VAS, 0–10)	11 years	Thenar eminence of the non-dominant hand
Walker et al. (43)	Retrospective observational cohort study	United Kingdom	*N* = 150 (102 preterm GA: 24.9 ± 0.8 weeks; 48 term-born)	Thermal test: HPT (^o^C), WDT (^o^C), CPT (^o^C), CDT (^o^C)	18–20 years	Thenar eminence of the non-dominant hand,
				Mechanical test: MDT (vFh), MPT (mN), PPT (kPa), Brush allodynia (VAS, 0–10)		
Hermann et al. (44)	Retrospective observational cohort study	Germany	*N* = 59 (19 preterm NICU GA: 28.6 ± 2.0 weeks; 20 term-born NICU; 20 term-born)	Thermal test: HPT (^o^C), *Δ*T (^o^C) Mechanical test: MPT (mN), MPS (NRS, 0–100)	9–14 years	Thenar eminence of the non-dominant hand and Maxillary branch of trigeminal nerve
Iversen et al. (38)	Prospective observational cohort	Norway	*N* = 203 (51 preterm GA: 28.8 ± 2.6 weeks; 66 term-born small for gestational age; 86 term-born)	Thermal test: HPT (^o^C), WDT (^o^C), CPT (^o^C), CDT (^o^C).	28 years	Volar wrist, anterior tibia, volar forearm, finger tip
				Mechanical test: PPT (kPa), Supra-threshold heat pain response test (NRS, 0–10)		
Hohmeister et al. (26)	Retrospective observational cohort study	Germany	*N* = 27 (9-preterm NICU GA: 28.6 ± 2.0 weeks; 9 term-born NICU; 9 term-born)	Thermal test: HPT (^o^C), WDT (^o^C), *Δ*T (^o^C)	11–16 years	thenar eminence of the left hand

CDT, Cold Detection Threshold; CPT, Cold Pain Threshold; HPT, Heat Pain Threshold; GA, Gestational Age; KPa, kilopascals; MDT, Mechanical Detection Threshold; mN, milliNewton; MPS, Mechanical Perceptual Sensitization; NICU, Neonatal Intensive Care Unit; NRS, Numeric Rating Scale; Thermal Perceptual Sensitization, *Δ*T; VAS, Visual analogue scale; vFh, Von Frey hair number; WDT, Warm Detection Threshold.

### 3.3 Methodological quality

**Figure 2:**
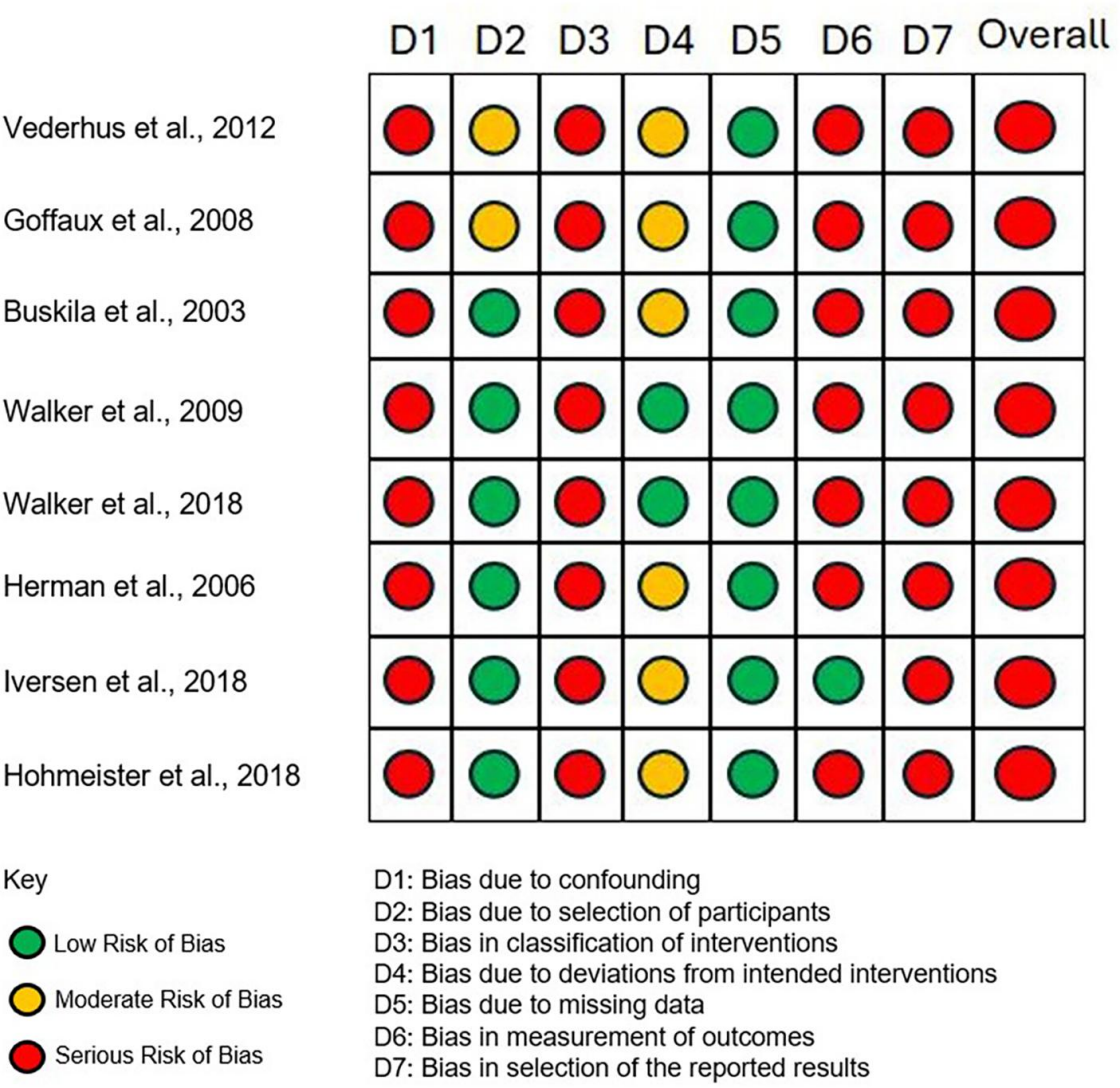
Summarizes the risk of bias across seven domains (D1–D7) for all eight studies. Overall, most studies were judged to have a serious risk of bias, primarily due to limitations inherent in retrospective designs.

The risk of bias was evaluated independently by two reviewers (N.G-M and J.S-I) using the ROBINS-I tool for non-randomized studies (Figure 2). Agreement was reached on approximately 75% of the items. Most discrepancies were related to distinguishing between “unclear” and “high” risk of bias and were resolved through discussion with a third reviewer (S.H-I).

Figure 2 summarizes the assessment of risk of bias in the included studies. Overall, the studies showed a considerable risk of bias across several critical domains, particularly in the classification of exposure and in the measurement of outcomes, due to the use of retrospective medical records and the absence of blinding of the assessors. In fact, only one study reported that the assessor was blinded to group allocation (38), whereas in the others the presence of scars or the available clinical information could have influenced subjective pain ratings. In contrast, participant selection and missing data presented a low to moderate risk, as most studies used neonatal information recorded prior to outcome measurement and reported complete or nearly complete data. Moreover, the absence of previously registered protocols in all studies limited methodological transparency and was associated with a serious or unclear risk in the selection of the reported outcomes. Importantly, the overall serious risk of bias across all included studies reduces confidence in the pooled estimates. Therefore, although the meta-analytic findings suggest potential differences in pain processing between preterm-born and term-born individuals, these results should be interpreted cautiously.

### 3.4 Results of the quantitative analysis

A total of eight studies were included in the quantitative synthesis, covering six sensory and pain-related outcomes. These outcomes assessed pressure and thermal pain thresholds as well as thermal detection thresholds, providing a broad and integrated evaluation of whether preterm-born individuals differ from their term-born peers in nociceptive and somatosensory function.

**Figure 3:**
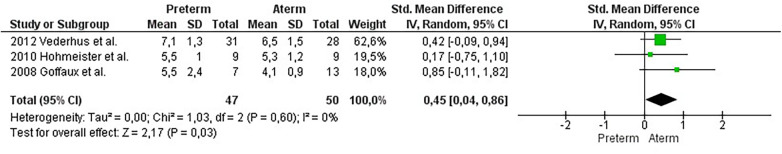
Standardized mean differences (SMD) comparing pain intensity between preterm and term-born participants. The forest plot displays the individual and pooled effects on pain intensity across studies. Squares represent the SMD for each study; diamonds represent the overall pooled effect. SMD, standardized mean difference; CI, confidence interval.

Across the three studies reporting pain intensity, which included 47 preterm and 50 term-born participants, the pooled effect showed a significant difference between groups (SMD = 0.45, 95% CI: 0.04 to 0.86, *p* = 0.03). According to Cohen's criteria, this represents a medium effect size. The forest plot showed lower pain intensity values in term-born controls compared with preterm-born participants (Figure 3), indicating higher self-reported pain intensity in the preterm group. Heterogeneity was not important (*I*^2^ = 0%).

**Figure 4:**
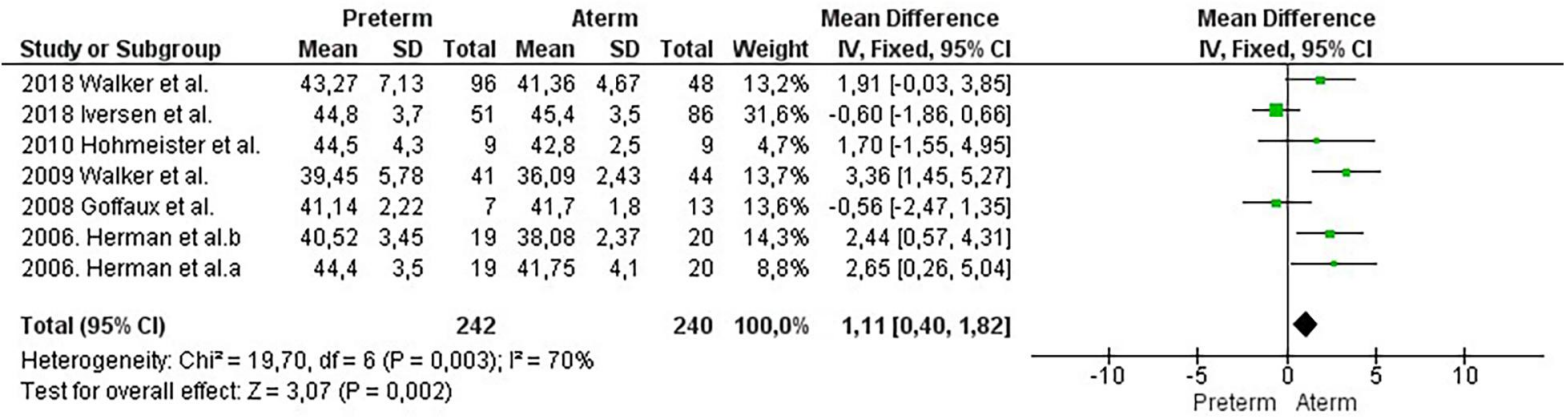
Mean differences (MD) comparing heat pain thresholds (HPT measured in °C) between preterm and term-born participants. The forest plot illustrates the individual and pooled effects for heat pain thresholds. Squares represent the MD for each study; diamonds indicate the pooled MD. MD, mean difference; SD, standard deviation; CI, confidence interval.

**Figure 5:**
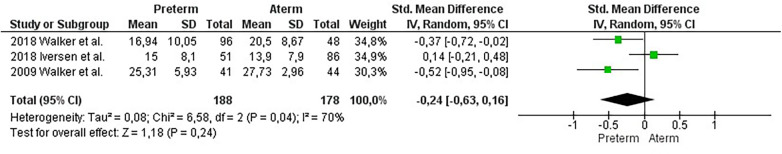
Standardized mean differences (SMD) comparing cold pain thresholds (CPT measured in °C) between preterm and term-born participants. Forest plot showing the SMD values for each study and the overall pooled estimate. SMD, standardized mean difference; CI, confidence interval.

Heat pain thresholds (HPT) were evaluated in seven studies involving 242 preterm and 240 term-born participants. The pooled mean difference revealed lower heat pain thresholds among term-born individuals (MD = 1.11, 95% CI: 0.40 to 1.82, *p* = 0.002), indicating greater heat pain sensitivity in this group. The forest plot illustrates this shift toward lower values among term-born participants (Figure 4). Heterogeneity was considerable (*I*^2^ = 70%). Cold pain thresholds (CPT), analyzed in three studies with 188 preterm and 178 term-born participants, did not show significant group differences (SMD = −0.24, 95% CI: −0.63 to 0.16, *p* = 0.24). This corresponds to a small effect size. The forest plot did not display a consistent shift toward either group (Figure 5). Heterogeneity was substantial (*I*^2^ = 70%).

**Figure 6:**
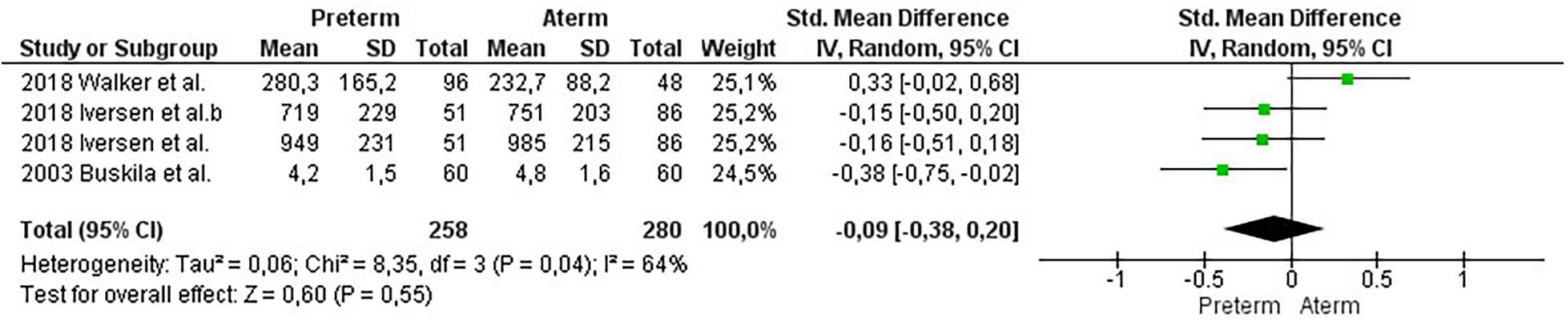
Standardized mean differences (SMD) comparing pressure pain threshold (PPT measured in kPa or kg/cm2) between preterm and term-born participants. The forest plot presents the effects on pressure pain threshold across study. Squares reflect the SMD for each study; diamonds show the pooled effect. SMD, standardized mean difference; CI, confidence interval.

Pressure Pain Threshold (PPT), assessed in four studies totaling 258 preterm and 280 term-born participants, also did not differ significantly between groups (SMD = −0.09, 95% CI: −0.38 to 0.20, *p* = 0.55). This represents a small effect size. The pooled estimate remained centered around the line of no effect (Figure 6). Heterogeneity was moderate to substantial (*I*^2^ = 64%).

**Figure 7:**
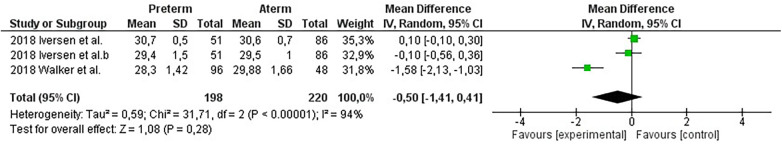
Mean differences (MD) comparing cold detection thresholds (CDT measured in °C) between preterm and term-born individuals. The forest plot displays the individual and pooled MD values for cold detection thresholds. D, mean difference; SD, standard deviation; CI, confidence interval.

**Figure 8:**
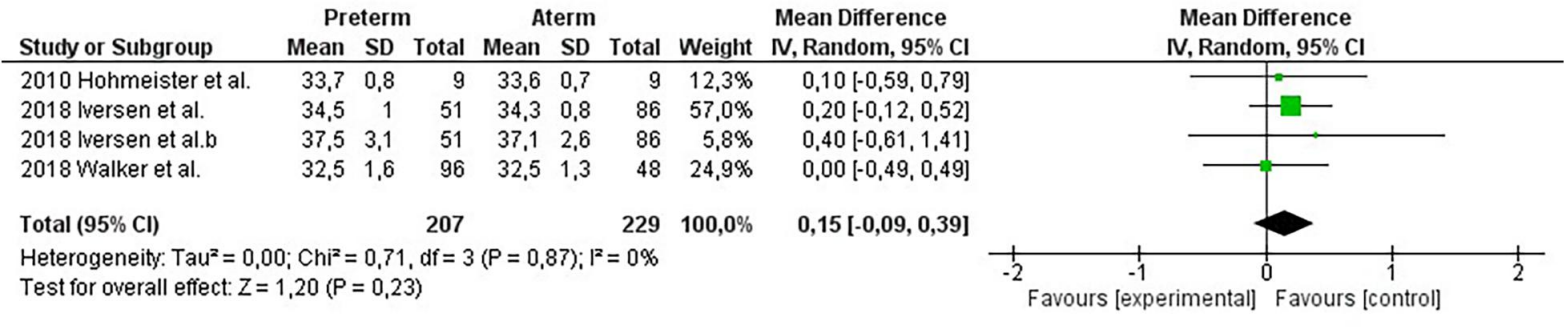
Mean differences (MD) comparing warm detection thresholds (WDT measured in °C) between preterm and term-born individuals. The forest plot summarizes the pooled term effects on warm detection thresholds. Squares represent MD per study; diamonds show the pooled MD. MD, mean difference; SD, standard deviation; CI, confidence interval.

Cold detection thresholds (CDT), examined in three studies involving 198 preterm and 220 term-born individuals, revealed no significant differences (MD = –0.50, 95% CI: −1.41 to 0.41, *p* = 0.28). The forest plot showed marked variability in both magnitude and direction of effects (Figure 7). Heterogeneity was considerable (*I*^2^ = 94%). Finally, warm detection thresholds (WDT), extracted from four studies including 207 preterm and 229 term-born participants, showed no significant differences between groups (MD = 0.15, 95% CI: −0.09 to 0.39, *p* = 0.23). The forest plot demonstrated balanced results with low heterogeneity (*I*^2^ = 0%) (Figure 8).

The GRADE assessment indicated very low certainty of evidence for all outcomes included in the meta-analysis. This was mainly due to the observational design of the included studies, the serious risk of bias identified across studies, the small number of studies contributing to several outcomes, and the presence of substantial or considerable heterogeneity and/or imprecision in some analyses (Supplementary Table S2). Taken together, preterm-born participants showed higher heat pain thresholds, suggesting reduced heat pain sensitivity in this specific modality. No clear group differences were observed for cold pain thresholds, pressure pain thresholds, or thermal detection thresholds. These findings suggest a modality-specific pattern mainly involving heat pain sensitivity, although further research is needed to confirm this interpretation. The presence of moderate, substantial, and considerable heterogeneity in several outcomes underscores the need for standardized assessment protocols and longitudinal designs. The presence of moderate, substantial, and considerable heterogeneity in several outcomes underscores the need for standardized assessment protocols and longitudinal designs to clarify the mechanisms underlying altered sensory processing in preterm-born individuals.

## 4 Discussion

The aim of this review was to analyze long-term data on thermal and mechanical pain thresholds, as well as pain intensity, in preterm-born individuals compared with those born at term, from childhood through young adulthood. The results of this systematic review and meta-analysis suggest that preterm-born individuals exhibit significantly altered pain thresholds compared to controls, and these differences may persist throughout childhood through young adulthood. However, the available evidence remains highly heterogeneous and shows notable methodological inconsistencies across studies. Therefore, these findings should be interpreted cautiously and considered as part of the growing body of evidence suggesting that preterm birth may have long-term effects on pain sensitivity.

Our analysis indicates that preterm-born individuals report higher pain intensity (NRS) than term-born controls, which is consistent with previous findings of increased self-reported pain sensitivity among early and moderately late preterm-born adolescents (45, 46). This result extends previous observations by confirming that the increase in pain intensity among preterm-born individuals is not limited to the neonatal or early childhood period but persists throughout childhood through young adulthood, suggesting that the nociceptive alterations initially documented in short-term studies (47–50), such as increased pain responses during heel lance, routine vaccinations, or other procedural pain tests in infancy, represent enduring changes in pain processing rather than transient phenomena. Moreover, longitudinal data indicate that a greater number of neonatal invasive procedures predicts higher self-reported pain intensity at school age in children born very preterm, independent of other clinical factors related to prematurity (23, 51).

Additionally, some studies suggest that preterm-born adolescents and young adults may also exhibit lower pain tolerance compared to term-born peers, although this outcome could not be meta-analysed due to the limited number of available studies. This supports the hypothesis of persistent alterations in pain modulation mechanisms following preterm birth (39, 46).

The results also show significantly higher heat pain threshold (HPT), in preterm-born children compared to those born at term. In contrast, the meta-analysis of cold pain threshold (CPT) did not reach statistical significance, despite two of the three included studies showing higher CPT values in preterm-born individuals. The lack of significance for CPT likely reflects the limited number of available studies and the heterogeneity in study design and measurement protocols, which may have reduced the statistical power to detect an effect. Nevertheless, the overall pattern across individual studies suggests a consistent directional trend toward thermal hypoalgesia. This finding suggests the presence of thermal hypoalgesia in the preterm-born population, which could reflect lasting alterations in the development of nociceptive pathways (22). Animal model studies support this hypothesis, as rats exposed to noxious stimuli during the neonatal period exhibited thermal hypoalgesia during development and into adulthood (52). In contrast, the study by Iversen et al. (38), which included young adults born with very low birth weight or small for gestational age, did not find significant differences in thermal pain thresholds compared to controls. This discrepancy could be explained by differences in the severity of prematurity, neonatal exposure to painful or surgical procedures, or length of stay in the NICU.

Importantly, the coexistence of higher pain intensity and elevated heat pain thresholds should not be interpreted as contradictory. Heat pain thresholds reflect the point at which a standardized thermal stimulus becomes painful, whereas pain intensity ratings reflect the perceived magnitude of pain once nociceptive activation has occurred and may be influenced by cognitive, emotional, and contextual factors. Furthermore, pain intensity was assessed using heterogeneous paradigms across the included studies, including suprathreshold heat pain, cold pressor pain, and self-reported pain experiences, limiting direct comparisons with heat pain thresholds. Experimental studies in formerly preterm individuals have suggested that neonatal pain exposure may be associated with reduced sensitivity to brief thermal stimuli while simultaneously promoting enhanced perceptual sensitization or altered pain modulation in response to more sustained or clinically relevant painful experiences (40, 44). Therefore, higher heat pain thresholds and greater pain intensity ratings may reflect different manifestations of altered pain processing rather than opposing findings. Overall, these modifications in thermal thresholds suggest alterations in the central modulation of nociceptive pathways, potentially reflecting plasticity processes induced by repeated exposure to pain and the immaturity of the descending inhibitory system during critical stages of development (13, 15). Taken together, these patterns, higher pain intensity and thermal hypoalgesia, point to complex and persistent changes in pain processing following preterm birth, likely driven by early-life nociceptive exposure and neurodevelopmental adaptations (53, 54). Such changes may indicate an exacerbated potentiation of central inhibitory mechanisms on thermal pain, with possible long-term repercussions for pain processing and response in adulthood, consistent with evidence linking preterm birth to chronic pain vulnerability (55). Notably, higher self-reported pain intensity can coexist with higher experimental heat pain thresholds, as these outcomes capture different dimensions of pain—subjective appraisal vs. modality-specific nociceptive processing.

However, no significant differences were detected between groups in heat and cold detection thresholds. This finding is consistent with previous quantitative sensory testing studies, which have shown that thermal detection thresholds and thermal pain thresholds assess partially distinct neurophysiological processes (56, 57). While thermal pain thresholds involve the activation of specific nociceptors and the central integration of nociceptive signals, processes that are highly modulated by peripheral and central sensitization phenomena as well as by descending inhibitory systems, thermal detection thresholds primarily reflect the basic sensory function of A*δ* and C fibers responsible for cold and heat perception, without the predominant involvement of complex nociceptive circuits (56–58). In this context, factors such as early and repeated exposure to painful stimuli, for example during NICU hospitalization, may preferentially alter central pain modulatory mechanisms, leading to changes in thermal pain thresholds without significantly affecting thermal detection.

Regarding pressure pain threshold, our meta-analysis did not show significant differences between preterm and term-born participants. The substantial heterogeneity observed across studies suggests considerable variability among the included data, likely reflecting differences in the mechanical stimuli used, testing procedures, and participant ages. All these sources of methodological inconsistency are well recognized in pediatric QST research (59, 60). Given the small effect size and the observed heterogeneity, the current evidence does not allow us to establish clear differences in mechanical pain sensitivity between preterm and term-born children.

### 4.1 Limitations and recommendations for future research

This systematic review and meta-analysis presents several limitations that should be considered when interpreting the findings. First, the limited number of included studies reduces statistical power and highlights the need for additional research in this area. Additionally, substantial heterogeneity was observed across studies, attributable to variability in methodological design, pain threshold measurement procedures, developmental stages assessed, anatomical regions analyzed, and, importantly, the number and type of clinical procedures performed during the neonatal period. The included studies also varied considerably regarding the neonatal characteristics of the preterm cohorts, including exposure to surgery, invasive procedures, NICU hospitalization, very low birth weight, and small-for-gestational-age status. Consequently, the observed differences in pain processing may reflect the combined influence of prematurity and associated neonatal factors. Future studies should aim to better characterize the independent contribution of these variables. Although several potential sources of heterogeneity were identified, formal subgroup analyses were not feasible because of the limited number of studies available for each outcome and the incomplete reporting of relevant clinical and methodological variables across studies. This heterogeneity complicates direct comparisons and limits the generalizability of the results. It should also be noted that in the present analysis, outcome data were available from a limited and heterogeneous set of anatomical sites (predominantly upper-limb locations, with some lower-limb assessments in a small number of studies), which restricts extrapolation to other body regions. Another relevant limitation is the lack of sex-stratified analyses in most studies, preventing exploration of potential sex-related differences. Furthermore, the methodological quality of the studies included was low: assessment using the ROBINS-I tool indicated a serious risk of bias in all studies, with only one employing assesor blinding (38). These limitations reduce confidence in the pooled estimates and indicate that the findings should be interpreted cautiously as exploratory and hypothesis-generating rather than definitive. A sensitivity analysis excluding studies at the highest risk of bias was considered; however, this was not feasible because all included studies presented serious risk of bias in at least one domain and several outcomes were informed by only a small number of studies. Excluding these studies would have substantially reduced the available evidence and, for some outcomes, would have prevented meaningful pooled analyses. Additionally, none of the studies reported a predefined analysis plan or referenced a registered protocol, which increases the risk of bias due to lack of transparency and methodological planning. Therefore, future research should adopt more rigorous designs, including blinding strategies, prior protocol registration, and bias control measures, to confirm and expand upon these findings.

Further research is essential to clarify these results and elucidate the underlying mechanisms. A deeper understanding of long-term sensory alterations in preterm-born individuals could inform the development of improved neonatal pain management strategies and guide care practices aimed at minimizing adverse outcomes later in life. Overall, these findings underscore the need for standardized approaches to pain assessment in preterm-born populations and longitudinal studies to determine whether early-life alterations in pain thresholds persist across developmental stages.

### 4.2 Practical implications for nursing

For clinical nursing practice, these findings emphasize the importance of adopting a proactive, developmentally sensitive approach to pain assessment and management in preterm-born and formerly preterm-born populations across the lifespan. Nurses play a central role in minimizing painful experiences during neonatal hospitalization, and the evidence of persistent alterations in pain processing reinforces the need for the consistent use of validated, age-appropriate pain assessment tools, alongside strategies aimed at reducing cumulative pain exposure.

In neonatal settings, nursing practice should prioritize the clustering of procedures, anticipation of painful events, and systematic implementation of evidence-based comfort and analgesic interventions, including skin-to-skin contact, facilitated tucking, non-nutritive sucking, and sucrose analgesia when appropriate. These interventions, when applied consistently, may contribute not only to immediate pain relief but also to the modulation of early nociceptive experiences with potential long-term implications.

In pediatric and adolescent care settings, nurses should be aware that preterm-born individuals may exhibit atypical pain responses, including higher self-reported pain intensity and differences in thermal pain sensitivity. These findings support individualized pain assessment and careful interpretation of pain reports.

## 5 Conclusions

The findings of this systematic review and meta-analysis suggest that preterm-born individuals may be associated with persistent alterations in pain processing from childhood through young adulthood, reflected by higher pain intensity and elevated thermal pain thresholds, particularly for heat. These findings may reflect long-term differences in pain sensitivity associated with preterm birth and early-life experiences. Although no significant differences were found in thermal detection thresholds or pressure pain thresholds, the direction of the effects and the marked methodological heterogeneity limit interpretation and highlight the need for more rigorous and longitudinal studies including larger sample sizes and standardized assessment protocols. Overall, the evidence points to complex and enduring modifications in pain sensitivity following preterm birth, with important implications for optimizing neonatal pain management, guiding clinical interventions and understanding its later developmental consequences. Future research should also consider psychosocial factors, sex differences, and neuroimaging correlates to better elucidate underlying mechanisms and support personalized pain prevention strategies across development, incorporating long-term follow-up into clinical practice.

## Data Availability

The raw data supporting the conclusions of this article will be made available by the authors, without undue reservation.
