# Supplementary material for: Long-term alterations in pain sensitivity following preterm birth: a systematic review and meta-analysis
Source: Front Pediatr. 2026 Jul 8;14:1858823. doi: 10.3389/fped.2026.1858823 (PMC13388744; doi:10.3389/fped.2026.1858823)
Supplement: Supplementary file 1 [file Table1.docx]

| **Database** | **Search strategy** | **Results** |
| --- | --- | --- |
| Pubmed | ("Premature"[Mesh] OR "premature"[Title/Abstract] OR "preterm"[Title/Abstract] OR "neonatal intensive care"[Title/Abstract] OR "neonatal care units"[Title/Abstract]) AND ( ("Pain Measurement"[Mesh] OR"neonatal pain"[Title/Abstract] OR "painful procedures"[Title/Abstract] OR "pain exposure"[Title/Abstract] OR "neonatal stress"[Title/Abstract] OR "repetitive pain"[Title/Abstract] OR "early life pain"[Title/Abstract] OR "early life stress"[Title/Abstract] OR "neonatal pain experiences"[Title/Abstract]) AND ("term infants"[Title/Abstract] OR "fullterm*"[Title/Abstract] OR "adolescent"[Title/Abstract] OR "term*"[Title/Abstract] OR "fullterm children"[Title/Abstract] OR "pain threshold*"[Title/Abstract] OR "pain sensitivity"[Title/Abstract]) | 389 |
| WOS | ((("premature" OR "preterm*" OR "neonatal intensive care" OR "neonatal care units") AND ("neonatal pain" OR "painful procedures" OR "pain exposure" OR "neonatal stress" OR "repetitive pain" OR "repeated pain experiences" OR "early life pain" OR "early life stress" OR "neonatal pain experiences")) AND ("term infants" OR "fullterm*" OR "adolescent*" OR "term*" OR "fullterm children") AND ("pain threshold*" OR "pain sensitivity")) | 63 |
| Cochrane | (premature OR (preterm*) OR ("neonatal intensive care") OR ("neonatal care units") AND ("neonatal pain") OR ("painful procedures") OR ("pain exposure") OR ("neonatal stress") OR ("repetitive pain") OR ("repeated pain experiences") OR ("early life pain") OR ("early life stress") OR ("neonatal pain experiences") AND ("term infants") OR ("fullterm*") OR (adolescent) OR (term*) OR ("fullterm children") AND ("pain threshold*") OR ("pain sensitivity") | 139 |
| Scopus | ALL(premature OR preterm* OR "neonatal intensive care" OR "neonatal care units") AND ALL("neonatal pain" OR "painful procedures" OR "pain exposure" OR "neonatal stress" OR "repetitive pain" OR "repeated pain experiences" OR "early life pain" OR "early life stress" OR "neonatal pain experiences") AND ALL("term infants" OR "fullterm*" OR adolescent OR term* OR "fullterm children" OR "pain threshold*" OR "pain sensitivity") | 519 |

Supplementary table 1. Search strategies used in the databases. Detailed search equations employed in PubMed, Web of Science (WoS), Cochrane Library, and Scopus for the identification of studies
