# Supplementary material for: Long-term alterations in pain sensitivity following preterm birth: a systematic review and meta-analysis
Source: Front Pediatr. 2026 Jul 8;14:1858823. doi: 10.3389/fped.2026.1858823 (PMC13388744; doi:10.3389/fped.2026.1858823)
Supplement: Supplementary file 2 [file Table2.docx]

Supplementary Table 2. GRADE assessment of the certainty of evidence for pain-related outcomes in preterm-born versus term-born individuals

| **Outcome** | **No. studies** | **Risk of bias** | **Inconsistency** | **Indirectness** | **Imprecision** | **Publication bias** | **Certainty of evidence** | **Effect [95% CI]** | **Reasons for rating** |
| --- | --- | --- | --- | --- | --- | --- | --- | --- | --- |
| Pain intensity | 3 | Serious | Not serious | Not serious | Serious | Not assessed | Very low | SMD = 0.45 [0.04, 0.86] | Observational evidence; serious risk of bias; small number of studies |
| Heat pain threshold | 7 | Serious | Serious | Not serious | No | Not assessed | Very low | MD = 1.11 [0.40, 1.82] | Observational evidence; serious risk of bias; substantial heterogeneity |
| Cold pain threshold | 3 | Serious | Serious | Not serious | Serious | Not assessed | Very low | SMD = −0.24 [−0.63, 0.16] | Observational evidence; serious risk of bias; substantial heterogeneity; imprecision |
| Pressure pain threshold | 4 | Serious | Serious | Not serious | Serious | Not assessed | Very low | SMD = −0.09 [−0.38, 0.20] | Observational evidence; serious risk of bias; substantial heterogeneity; imprecision |
| Cold detection threshold | 3 | Serious | Very serious | Not serious | Serious | Not assessed | Very low | MD = −0.50 [−1.41, 0.41] | Observational evidence; serious risk of bias; considerable heterogeneity; imprecision |
| Warm detection threshold | 4 | Serious | Not serious | Not serious | Serious | Not assessed | Very low | MD = 0.15 [−0.09, 0.39] | Observational evidence; serious risk of bias; imprecision |
| GRADE: Grading of Recommendations Assessment, Development and Evaluation; SMD: standardized mean difference; MD: mean difference; CI: confidence interval.  Risk of bias: rated as serious when the contributing studies were judged to have serious risk of bias according to ROBINS-I. Inconsistency: rated as serious when I² >40% and very serious when I² >80%. Indirectness: rated as not serious when population, exposure, comparator, and outcomes directly matched the review question. Imprecision: rated as serious when the number of studies was small and/or the 95% CI crossed the line of no effect. Publication bias: not assessed because fewer than ten studies were available for each outcome. Quality of evidence: observational evidence started as low certainty and was downgraded when serious concerns were identified. | | | | | | | | | |
